# Biocompatibility Evaluation of Porcine-Derived Collagen Sheets for Clinical Applications: In Vitro Cytotoxicity, In Vivo Sensitization, and Intracutaneous Reactivity Studies

**DOI:** 10.3390/jfb16090347

**Published:** 2025-09-15

**Authors:** Tae-Hoon Koo, Jason K. Lee, Shawn P. Grogan, Darryl D. D’Lima

**Affiliations:** 1 D.med LLC, 111, Sagimakgol-ro, Jungwon-gu, Seongnam-si 13202, Gyeonggi-do, Republic of Korea; glenkoo@dmed.co.kr; 2 Shiley Center for Orthopaedic Research and Education at Scripps Clinic, 3550 John Hopkins Ct, Suite 110, San Diego, CA 92121, USA; sgrogan@scripps.edu

**Keywords:** porcine collagen, biocompatibility, cytotoxicity, skin sensitization, intracutaneous reactivity, biomedical materials

## Abstract

Biocompatibility evaluation of medical devices is essential for ensuring safety, with ISO 10993 series being the standard. However, these tests can be time-consuming and resource-intensive. This study assessed the early-stage biocompatibility of a collagen matrix derived from porcine dermis using three selective ISO tests: in vitro cytotoxicity, in vivo sensitization, and irritation. Collagen was hydrolyzed, purified from miniature pig skin, and then processed into porous sheets via lyophilization. Extracts were prepared using both polar and non-polar extraction vehicles for biological testing. Cytotoxicity testing with mouse fibroblast cells showed no significant cytotoxic effects, with cell morphology and viability comparable to controls. Sensitization testing in guinea pigs, involving intradermal and topical exposure, revealed no allergic responses. Irritation testing in rabbits showed no signs of irritation. The cytotoxicity test took 3 days, the sensitization test 28 days, and the irritation test 7 days, all of which proved suitable for early biocompatibility screening. These results confirmed that the collagen matrix is non-cytotoxic, non-sensitizing, and non-irritant for a month. The use of these three tests enables early identification of unsafe materials, reducing time, cost, and animal use before advancing to more complex ISO 10993 studies. Therefore, they are appropriate and necessary for early feasibility decisions in medical device development.

## 1. Introduction

Porcine dermal collagen matrix has emerged as a widely used biomaterial in clinical applications due to its structural and biochemical similarity to human extracellular matrix (ECM) proteins [1,2,3]. These properties allow it to support cellular adhesion, modulate biological signaling, and facilitate tissue regeneration, making it valuable for wound care, tissue engineering, surgical procedures, and regenerative medicine [4,5,6,7,8].

Despite its broad clinical use and general biocompatibility, rigorous safety evaluations remain essential, particularly when developing new formulations or applications, due to potential immune responses associated with collagen derivatives or denatured collagen [9,10,11]. The production of clinically usable collagen from animal tissues involves a multi-step process to ensure the final product meets stringent safety and quality standards. This process includes raw material sourcing, extraction, hydrolysis, purification, formulation, packaging, and sterilization [12,13,14,15]. Given the critical nature of these products in medical settings, manufacturers prioritize sustainable sourcing and implement rigorous quality control throughout production. Leading collagen manufacturers often adhere to internationally recognized standards, such as Good Manufacturing Practices (GMP), to ensure the safety, purity, and consistency [16,17].

A broad range of collagen-based products is commercially available, including collagen peptides, gelatin, and hydrolyzed collagen, offered in various forms such as powders, capsules, and liquid solutions [18,19]. Among these, collagen sheets are particularly valued for their flexibility, porosity, and tunable physical and chemical properties, which make them ideal for diverse medical and clinical applications [7,20,21]. Engineered with varying degrees of thickness and porosity, collagen sheets can be tailored for applications in wound care, tissue repair, tissue augmentation, hemostasis, surgical procedures, and cosmetics [20,22,23,24].

Biocompatibility testing plays a foundational role in preclinical assessments, ensuring that materials do not cause cytotoxic, sensitizing, or irritant effects when in contact with human tissues [25]. The ISO 10993 series provides globally recognized standards for such evaluations [26,27,28]; however, full compliance testing can be time-consuming and resource-intensive, especially in the early development stage [29,30].

To support more efficient feasibility assessments, early-stage biocompatibility screening using selected ISO 10993 tests can help determine the initial safety of novel biomaterials before advancing to more complex and long-term evaluations. In this study, we assessed the biological safety of porcine dermis-derived collagen matrix sheets using three ISO 10993-compliant tests: in vitro cytotoxicity (ISO 10993-5), in vivo sensitization (ISO 10993-10), and irritation (ISO 10993-23). Collagen sheets were prepared from miniature pig skin through hydrolysis and lyophilization, and their short-term biocompatibility was evaluated using both polar and non-polar extract testing in cell and animal models.

This approach supports early decision-making during the medical device development by enabling the identification of potentially unsafe materials while minimizing time, cost, and animal use. The results of this study provide foundational data for advancing porcine dermal collagen matrices toward more comprehensive preclinical and clinical applications.

## 2. Materials and Methods

### 2.1. Preparation of Collagen Matrix, Testing Samples, and Designated Control Samples

**Figure 1:**
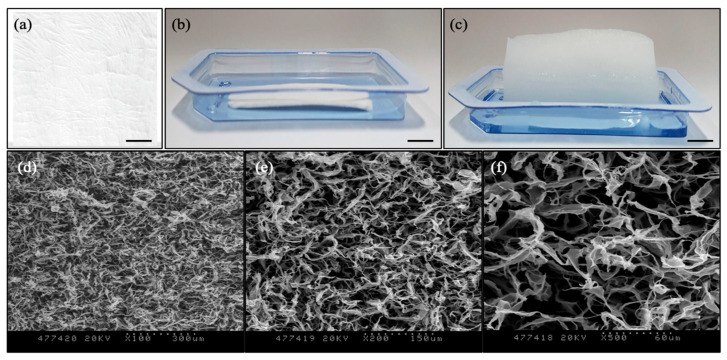
Porcine dermal collagen matrix: (**a**) Dense sheet type for covering; (**b**) Porous sheet type for tissue augmentation before rehydration; (**c**) Porous sheet type for tissue augmentation after rehydration (Scale bars: 1 cm). SEM images of porous collagen structure: (**d**) ×100 magnification (dotted scale bar: 300 μm); (**e**) ×200 magnification (dotted scale bar: 150 μm); (**f**) ×500 magnification (dotted scale bar: 60 μm).

Collagen matrix (D-med, Seongnam-si, Gyeonggi-do, Republic of Korea) was extracted and purified from specific pathogen-free miniature pig (SPF, 6 months old) skin as described earlier [31,32]. Briefly, 3% (*w*/*v*) collagen type I gel was extracted with hydrochloric acid, and the extracts were dissolved in PBS with pH adjusted between 6–8 with 10 M NaOH solution. The extracted gel was filtered with a 300 kDa cut-off size and a 100 kDa cut-off size sequentially. The purified collagen type I gel was placed into the rectangular-shaped mold and lyophilized to make a sheet. The collagen sheet was finally sterilized with ethylene oxide. The porous collagen sheet type was selected as a testing sample for the study (Figure 1).

All sample preparation and reference materials were prepared according to ISO 10993-12:2021 [33]. For the cytotoxicity test, high-density polyethylene film (HDPE film, Hatano Research Institute, Hadano, Kanagawa, Japan) was used as a negative control, and ZDEC polyurethane film (PU film, Hatano Research Institute, Hadano, Kanagawa, Japan) was used as a positive control. Minimum Essential Medium (MEM, Gibco, Waltham, MA, USA) with 10 % (*v*/*v*) horse serum (HS, Gibco, Waltham, MA, USA) and 1 % (*v*/*v*) penicillin-streptomycin (PS, Gibco, Waltham, MA, USA) was used for the extraction vehicle. The cells cultured with the extraction vehicle only were used for a media control. For the skin sensitization and the intracutaneous reactivity tests, sterile saline (Daihan Pharm, Seoul, Republic of Korea) was used as a polar extraction vehicle, and cottonseed oil (Junsei Chemical, Tokyo, Japan) was used as a non-polar extraction vehicle. The extraction vehicle, each of sterile saline only and cottonseed oil only, was used as a negative control for each extraction (by polar or non-polar extraction vehicle) of testing samples [33].

### 2.2. Cytotoxicity Test of Mouse Fibroblast Cells

A cytotoxicity test using the extraction testing method was conducted in accordance with ISO 10993-5: Test for in vitro cytotoxicity to qualitatively and quantitatively assess whether the collagen sheet releases any soluble substances that may induce cytotoxic effects [26]. Each collagen sheet test sample was extracted in the extraction vehicle, MEM media (3 cm^2^/mL, n = 3), with gentle shaking at 37 °C for 24 h in a 5% CO_2_ incubator. Negative (HDPE film) and positive controls (PU film) were extracted in MEM media (0.1 g/mL, n = 3) under the same conditions. The extracts from the testing sample, negative control, and positive control material were stored at room temperature and used for the test within 4 h. Then, 2 mL of mouse fibroblast cells (10^5^ cells/mL, L-929, ATCC, Manassas, VA, USA) was plated on the 6-well plate and maintained for more than 24 h. Two milliliters of extract from each sample (testing material, negative control, and positive control) was added to the maintained cells in the well. After the extract’s inoculation, the cell plate was incubated at 37 °C, 5% CO_2_ for 48 h. Afterward, cytomorphological changes and lysis of each cell were observed microscopically based on the cytotoxicity assessment criteria [26] (Table 1).

After the morphological grading, the number of viable cells was counted after detaching the cells with trypsin (Gibco, Waltham, MA, USA) treatment, and the detached cells were counted with a hematocytometer after the cell staining with Trypan Blue (Gibco, Waltham, MA, USA) [34]. The relative cell count of the testing sample group was calculated as a percentage of the cell number of the media control group as below.

(1) $$\mathrm{RCC}\;(\mathrm{Relative}\;\mathrm{cell}\;\mathrm{counting},\;\%)=\frac{Cell\;number\;of\;test\;group}{Cell\;number\;of\;media\;control\;group}\times 100$$

### 2.3. Sensitization Test of Guinea Pigs

The animal study was approved by the Institutional Animal Care and Use Committee (IAC20222931, Korea Testing & Research Institute, Jeollanam-do, Republic of Korea). A total of 30 Dunkin Hartley Guinea Pigs (male, 5 weeks, Samtako Bio, Osan, Republic of Korea) were studied. The test was to investigate the skin sensitizing potency of the collagen sheet using its polar and non-polar vehicle extract according to ISO 10993-10: Tests for skin sensitization [27] (Table 2).

**Figure 2:**
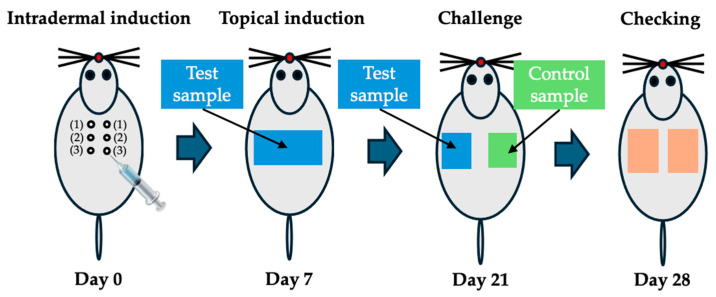
Location of intradermal injection (0.1 mL) sites in guinea pigs (n = 5: control, n = 10: test) at intradermal induction phase (Day 0). Filter paper containing the test samples is attached at topical induction phase (Day 7). Filter paper containing test samples and control samples is replaced at challenge phase (Day 21). Skin reactions are evaluated on the application site after removing the filter paper at checking phase (Day 28).

A testing sample with an extraction vehicle (6 cm^2^/mL, n = 5) was incubated at 37 °C for 72 h with gentle agitation. After centrifugation at 3000 rpm for 10 min, the collected extracts were filtered with a 0.45 μm filter. The intrascapular (shoulder) region of each guinea-pig was shaved to expose an area of approximately 4 cm × 6 cm. Extract (0.1 mL) was injected intradermally into the dorsal scapular region for the intradermal induction phase (Figure 2).

Five days after the intradermal induction phase, the fur on the intradermal injection area was clipped off, and the area was treated with 10% Sodium Dodecyl Sulfate (SDS, Bioneer, Seoul, Republic of Korea), 0.5 mL. On the following day (24 h), filter papers (2 cm × 4 cm) containing the test samples (0.4 mL) were prepared on a non-irritating tape (Tegarderm, 3M, Saint Paul, MN, USA). It was attached to the intradermal induction area of each animal and held using a Coban bandage (Coban, 3M, Saint Paul, MN, USA) for 48 h. In the same way, control samples (0.4 mL) were treated in control animals for the topical induction phase. At 13 days after completion of the topical induction phase, a filter paper (2 cm × 2 cm) containing a test sample (on the left side, 0.2 mL) and a control sample (on the right side, 0.2 mL) was prepared, and attached to the non-irritant tape. The tape was attached to the upper flank area of each animal and held using a Coban bandage for 24 h for the challenge phase. The skin reaction was evaluated on the application site at 24–48 h after the removal of the patch. The skin response was evaluated and graded according to the assessment scale for skin reaction [27] (Table 3). The positive control test was conducted every 6 months using DNCB (1-chloro-2,4-dinitrobenzene) under the ISO 10993-10 [27].

### 2.4. Irritation Test of Rabbits

**Figure 3:**
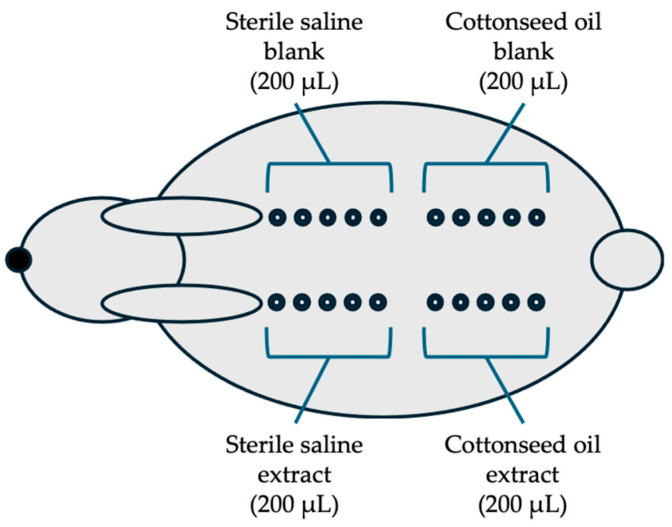
Arrangement of the injection sites in rabbits: The five injections (200 μL each) of each group (saline extract, saline blank, cottonseed extract, and cottonseed blank) are on one side of the spine of each rabbit (n = 3) and observed at 24, 48, and 72 h after injection.

The animal study was approved by the Institutional Animal Care and Use Committee (IAC20222665). A total of 3 New Zealand white rabbits (male, 3 months, DooYeol Biotech, Seoul, Republic of Korea) were used. The test aimed to evaluate the intracutaneous response to extracts of the collagen sheet in the rabbits following intracutaneous injection according to ISO 10993-23: Test for irritation [28]. A testing sample with either sterile saline as a polar extraction vehicle or sterile cottonseed oil as a non-polar extraction vehicle, an extraction vehicle (6 cm^2^/mL, n = 4), was incubated at 37 °C for 72 h with gentle agitation. After centrifugation at 3000 rpm for 10 min, the collected extracts were filtered with a 0.45 μm filter. Then, 200 μL of the test sample extracts of the sterile saline and cottonseed oil were injected intracutaneously at each of the five sites on one side of the spine of each rabbit using a 1 mL syringe with a 25-gauge needle (Figure 3).

Two hundred microliters of negative controls were injected at five sites of the contralateral side of each rabbit. The appearance of each injection site was observed immediately after injection and at 24, 48, and 72 h after injection. The injection sites were scored for erythema and edema according to the system (Table 4). The positive control test was conducted every 6 months using 0.5% SDS according to ISO 10993-23 [28].

### 2.5. Acceptance Criteria of Test Grading System

In the quantitative cytotoxicity test, when the relative cell count is 70% or greater, the test result is generally considered a passing level for cytotoxicity testing. The cytotoxicity assessment system for the qualitative cytotoxicity test analysis is met if the grade is 2 or less. The skin reaction scale results for the sensitization test are met if the grades of 1 or greater in the test group, generally indicating sensitization, provided that grades of less than 1 are seen in the control animals. The scoring system for irritation reactions is met if the difference between the test extracts and the negative control overall mean score is 1 or less.

## 3. Results

### 3.1. Cytotoxicity Test

**Figure 4:**
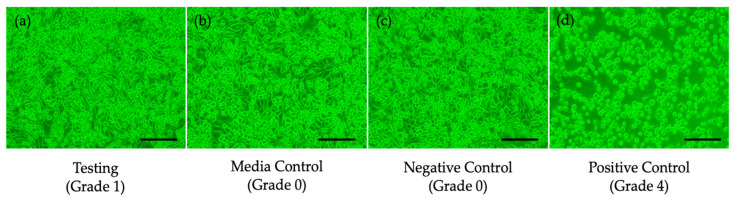
Microscopy of cell morphology at 24 h after treatment: (**a**) testing sample extract-treated cells (Grade 1), (**b**) medical control (Grade 0), (**c**) negative control (Grade 0), (**d**) positive control (Grade 4, scale bar: 50 μm).

After the extraction, the extracts with the collagen sheet appeared clear and free of particulates and retained a reddish-orange color in the medium. In the cytotoxicity test using L-929 cells, 10% of the cells were observed to be round and loosely attached. The cell morphology of the media control (cells with MEM culture media only) verified that the culture media (extraction vehicle) did not impact cytotoxicity during the test (Figure 4).

The relative cell counting of the test group revealed 92.0% viability, and the cytotoxicity reactivity grade was assessed as Grade 1, indicating that the cytomorphology showed only slight growth inhibition and slight reactivity in the testing sample group (Table 5). Both results indicate no critical cytotoxic effects from the collagen sheet, with only minimal reactivity observed. Therefore, the collagen sheet is considered biocompatible and suitable for subsequent animal studies.

### 3.2. Skin Sensitization Test

**Figure 5:**
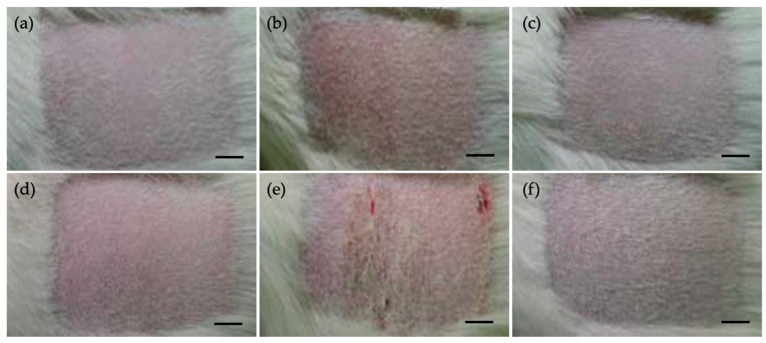
Photograph of individual skin reaction in Dunkin Hartley Guinea pigs. (**a**–**c**) 24 h after challenge, (**d**–**f**) 48 h after challenge, (**a**,**d**) negative control, (**b**,**e**) positive control, (**c**–**f**) test sample extract-treated animals (scale bar: 1 cm).

No test sample-related clinical signs, mortality, or other adverse effects were observed during the observation period in Guinea Pigs. No significant changes in body weight were noted following administration of the test sample. Evaluation of skin responses at 24 and 48 h after the challenge showed no evidence of erythema, edema, or any other skin reactions in either the control or test groups (Figure 5, Table 6).

Sensitization scores for both the polar vehicle test group (G2) and the non-polar vehicle test group (G4) remained at baseline levels at both time points, indicating no sensitization response. Based on these results, the collagen sheet is considered to have no skin sensitization potential as assessed by the Guinea Pig maximization test.

### 3.3. Irritation Test

All erythema and edema grades observed in in New Zealand white rabbits at 24, 48, and 72 h were combined separately for each animal. The total grading for each animal was divided by 15, corresponding to the three scoring time points and five test injection sites. Subsequently, the mean score for each solution was determined by dividing the total grade by 3. The negative control was assessed using the same calculation.

The test was considered successful if the difference between the overall mean scores of the test extracts and the negative control did not exceed 1.0. Throughout the study, no clinical signs related to the test samples or animal deaths were observed. Local reactions included very slight erythema and well-defined erythema at injection sites for the sterile saline extracts, and very slight erythema at sites injected with cottonseed oil extracts. The calculated differences between the test sample and negative control were 0.78 for the sterile saline extracts and 0.00 for the cottonseed oil extracts (Table 7).

Thus, for each extract of the collagen sheet in rabbits, the difference between the test sample and negative control was 1.0 or less, confirming that the requirements of the intracutaneous reactivity test were met.

## 4. Discussion

In this study, we evaluated the early-stage biocompatibility of a porcine dermal collagen matrix using a series of ISO 10993-compliant tests, including in vitro cytotoxicity, in vivo skin sensitization, irritation, and intracutaneous reactivity assays. These tests represent foundational components in the biological evaluation of medical devices, particularly those intended for contact with skin or soft tissues [35]. The cytotoxicity test, conducted using L-929 fibroblast cells, demonstrated that the collagen sheet exhibited a minimal cytotoxic response (Grade 1), with relative cell viability at 92.0%. According to ISO 10993-5, a material with a reactivity grade 2 or less and cell viability 70% or greater is considered non-cytotoxic [26]. The primary endpoints of the ISO 10993-5 test include cell viability, morphological changes, cell detachment, and cell lysis, enabling the assessment of both cell viability and potential adverse cellular reactions. These results align with previous studies showing that porcine-derived collagen materials generally support cellular viability and proliferation due to their biochemical similarity to human extracellular matrix (ECM) proteins [3,36].

The skin sensitization test, conducted using the guinea pig maximization method as per ISO 10993-10, revealed no evidence of erythema, edema, or other hypersensitivity reactions, indicating that the collagen sheet lacks sensitizing potential. Sensitization testing is essential for assessing medical devices for potential allergic or hypersensitivity reactions. Its purpose is to determine whether a device can sensitize the immune system, leading to allergic responses upon subsequent exposures. The results are consistent with existing literature reporting that porcine collagen exhibits low immunogenicity in dermal applications [37,38]. Notably, although collagen is a xenogeneic biomaterial, purification and processing steps such as enzymatic hydrolysis and lyophilization are known to significantly reduce antigenicity [7,39].

In the intracutaneous reactivity test, the difference in irritation scores between test control groups remained below the ISO 10993-23 threshold of 1.0, confirming that the collagen extracts do not induce significant local tissue irritation. This testing method is crucial for identifying any irritating properties of medical device extracts. Similar findings have been reported in studies of collagen-based wound dressing and scaffolds, where localized tissue responses were minimal or absent in rabbit and rodent models [22]. Collectively, these results support the biocompatibility of the porcine dermal collagen matrix in contact with skin or soft tissue. The successful outcomes in these tests provide a sound basis for progressing to more advanced assessments, such as implantation, subchronic toxicity, and systemic immunogenicity studies, which are essential for evaluating long-term safety and functional integration.

Collagen, a major component of the extracellular matrix, contains specific integrin-binding domains that facilitate cellular adhesion [40,41]. In addition to serving as a physical scaffold, collagen actively interacts with cell surface receptors, modulating cellular behavior and influencing key signaling pathways, including those involving growth factors [40,42]. Due to its excellent biocompatibility and versatility, collagen is widely utilized in biomedical applications in various physical forms, including sheets, gels, powders, and pastes [43,44]. Specifically, collagen sheets are flat, flexible layers with varying porosities, making them ideal for wound management, surgical procedures, soft tissue augmentation, and hemostasis.

Biocompatibility testing is a fundamental component of the preclinical evaluation of medical devices, playing a pivotal role in ensuring their safety and efficacy prior to clinical use [25]. While in vitro toxicity tests are valuable, they have limitations when used in isolation for evaluating newly developed medical devices [45]. Moreover, the in vitro tests still present technical challenges in fully incorporating with regulatory-compliant biocompatibility evaluations [46,47,48]. The three biocompatibility tests conducted in this study effectively identified potential biological risks, including toxicity, cytotoxicity, irritation, or allergic responses in early stages of evaluation, meeting essential minimum requirements.

Given the significant health implications associated with medical devices, regulatory authorities such as the Food and Drug Administration (FDA) and the European Medicines Agency (EMA) mandate comprehensive biocompatibility evaluations. These assessments are critical for compliance with established safety standards before any clinical application [49]. One of the primary guidelines for conducting biocompatibility testing is the ISO 10993 standard, which provides a systematic approach to the biological evaluation of medical devices. This international standard outlines a series of tests designed to assess the potential interactions between the device materials and the biological system, ensuring that the device does not induce adverse biological effects [50].

Although animal-derived collagen offers several biological advantages, its use raises concerns related to immunogenicity, pathogen transmission, contamination, and environmental sustainability [51]. These issues underscore the need for continued safety evaluations and risk-benefit analyses, particularly as medical devices advance toward long-term or implantable applications. Overall, the early biocompatibility assessments of cytotoxicity, sensitization, and irritation conducted in this study provide an effective foundation for determining the biological safety of a new medical device, facilitating its transition to more complex evaluations prior to clinical use.

## 5. Conclusions

This study demonstrated that porcine-derived collagen sheets are non-cytotoxic, non-sensitizing, and non-irritant based on selective ISO 10993 biocompatibility tests. The successful outcomes of in vitro cytotoxicity, in vivo sensitization, and intracutaneous reactivity tests suggest that these collagen sheets are safe for early-stage biomedical applications. These three assays proved effective for rapid, resource-efficient screening, allowing for early identification of unsuitable materials and minimizing unnecessary animal use. While the results are promising, further comprehensive biocompatibility assessments, including long-term and functional evaluations, are required to confirm the material’s suitability for clinical use. Overall, the findings support the continued development of porcine collagen sheets as potential candidates for medical device applications.

## Figures and Tables

**Table 1:** Cytotoxicity assessment criteria.

Graph	Level of Reactivity	Description of Cell Culture Conditions
0	No Reaction	Cells maintain normal morphology with occasional intracellular granules; no visible damage or inhibition of cell proliferation
1	Minimal Reaction	Up to 20% of cells appear rounded or loosely attached with minor morphological changes or granule loss; few lysed cells; cell growth largely unaffected.
2	Mild Reaction	Up to 50% of cells exhibit rounding, granule loss, or early signs of detachment; minimal cell death; moderate reduction in cell growth may be observed.
3	Moderate Reaction	Between 50–70% of the culture displays cell rounding, detachment, or lysis; significant inhibition of cell proliferation, through some intact cell layers remain.
4	Strong Reaction	Most or all of the cell layer is disrupted or destroyed; widespread cell death and major loss of cell integrity.

**Table 2:** Intradermal injection solution by group.

Group	Animal No.	Test Materials	Dose (mL)	Injection Site
G1	5	Sterile saline: * FCA (1:1) Sterile saline Sterile saline: ** FCA emulsion (1:1)	0.1 0.1 0.1	(1) (2) (3)
G2	10	Sterile saline: * FCA (1:1) Sterile saline extract Sterile saline extract: ** FCA emulsion (1:1)	0.1 0.1 0.1	(1) (2) (3)
G3	5	Cottonseed oil: * FCA (1:1) Cottonseed oil Cottonseed oil: ** FCA emulsion (1:1)	0.1 0.1 0.1	(1) (2) (3)
G4	10	Cottonseed oil: * FCA (1:1) Cottonseed oil extract Cottonseed oil extract: ** FCA emulsion (1:1)	0.1 0.1 0.1	(1) (2) (3)

* FCA (Freund’s Complete Adjuvant): The composition is a mixture of a non-metabolizable oil, a surfactant, and mycobacteria. It plays a crucial role in stimulating a strong and prolonged immune response to the test material (antigen). ** FCA emulsion: The prepared mixture of FCA and antigen for testing. It delivers the antigen and plays a critical role in enhancing and driving the sensitization process.

**Table 3:** Assessment scale for skin reaction.

Observed Reaction	Score
No visible skin change	0
Slight redness in localized areas	1
Noticeable, widespread redness	2
Pronounced redness and/or swelling	3

**Table 4:** Scoring criteria for intracutaneous irritation reactions.

Reaction Type	Score	Description
**Skin Redness (Erythema)**		
**None**	0	No observable redness
Slight	1	Faint or minimal redness, just detectable
Clear	2	Noticeable redness with clear borders
Marked	3	Pronounced redness over the area
Severe	4	Deep red coloration or formation of scab masking redness
**Swelling (Edema)**		
None	0	No visible swelling
Slight	1	Barely noticeable swelling
Clear	2	Obvious swelling within the treated area
Moderate	3	Raised skin, approximately 1 mm above surface
Severe	4	Pronounced swelling exceeding 1 mm and spreading beyond treated area

**Table 5:** Cytotoxicity quantitative and qualitative analyses.

Viable Cell Count	Test	Media Control	Negative Control	Positive Control
1	7.9 × 10^5^	8.3 × 10^5^	8.1 × 10^5^	0
2	8.2 × 10^5^	8.9 × 10^5^	8.3 × 10^5^	0
3	7.8 × 10^5^	8.7 × 10^5^	8.4 × 10^5^	0
Average	8.0 × 10^5^	8.7 × 10^5^	8.3 × 10^5^	0
RCC (%)	92.0	100.0	95.4	0.0
Grade	1	0	0	4

**Table 6:** Evaluation of the skin response in Dunkin Hartley Guinea pigs.

Graph 24.	24 h			48 h		
	Negative Control	Positive Control	Test	Negative Control	Positive Control	Test
Average	0.00	1.40	0.00	0.00	1.70	0.00
SD	0.00	0.52	0.00	0.00	0.48	0.00

**Table 7:** Irritation reaction results in New Zealand white rabbits.

Animals	Saline Extracts		Cottonseed Oil Extracts	
	Test	Control	Test	Control
1	1	0	0.67	0.67
2	0.33	0	0.33	0.33
3	1	0	0.67	0.67
Average *	0.78	0.00	0.56	0.56
Score difference **	0.78		0.00	

* Average: Erythema and Edema scores of each injection site were assessed at 24, 48, and 72 h time points, and the accumulated scores per animal were averaged. ** Score difference: Treatment irritation score—Control irritation score.

## Data Availability

The data that support the findings of this study are available from the corresponding author upon reasonable request.

## Abbreviations

ISO	International Organization for Standardization
MEM	Minimum essential medium
HS	Horse serum
PS	Penicillin streptomycin
ATCC	American Type Culture Collection
IAC	Institutional Animal Care and Use Committee
RPM	Revolutions per minute
SDS	Sodium dodecyl sulfate
DNCB	2,4-Dinitrochlorobenzene
